# *Lactiplantibacillus plantarum* NMGL2 exopolysaccharide ameliorates DSS-induced IBD in mice mainly by regulation of intestinal tight junction and NF-κB p65 protein expression

**DOI:** 10.3389/fmicb.2024.1491727

**Published:** 2024-10-28

**Authors:** Zengjia Zhou, Min Zhang, Mengke Yao, Jasra Naseeb, Abid Sarwar, Zhennai Yang, Tariq Aziz, Majid Alhomrani, Walaa F. Alsanie, Abdulhakeem S. Alamri

**Affiliations:** ^1^Key Laboratory of Geriatric Nutrition and Health of Ministry of Education, Beijing Engineering and Technology Research Center of Food Additives, Beijing Technology and Business University, Beijing, China; ^2^Key Laboratory of Agro-Products Primary Processing, Academy of Agricultural Planning and Engineering, MARA, Beijing, China; ^3^Institute of Molecular Biology and Biotechnology, The University of Lahore, Lahore, Pakistan; ^4^Department of Clinical Laboratory Sciences, The Faculty of Applied Medical Sciences, Taif University, Taif, Saudi Arabia

**Keywords:** dextran sulfate sodium, *Lactiplantibacillus plantarum*, inflammatory bowel disease, exopolysaccharide, NF-κB

## Abstract

Treatment of inflammatory bowel disease (IBD), a common chronic intestinal disease, by exopolysaccharides (EPSs) produced by lactic acid bacteria has raised increasing concerns. Here, the EPS produced by *Lactiplantibacillus plantarum* NMGL2 was evaluated for its ameliorating effect on dextran sodium sulfate (DSS)-induced IBD in mice. Administration of the EPS was shown to decrease the body weight loss and the values of disease activity index (DAI) and alleviate the colon damage as evidenced by an improvement in colonic length shortening, a reduction in colonic coefficient, and a reduction in colonic mucosal architecture and inflammatory infiltration. Cytokine assay of the blood and colon tissue samples showed that the EPS could decrease the levels of pro-inflammatory TNF-α and IL-1β, and increase anti-inflammatory IL-10. Oxidative stress assay of the colon tissue showed that the nitric oxide (NO) and malondialdehyde (MDA) levels decreased significantly (*p* < 0.05), while superoxide dismutase (SOD) and glutathione (GSH) levels increased significantly (*p* < 0.05) after the EPS intervention. These results were further confirmed by the significantly (*p* < 0.05) down-regulated levels of NF-κB p65, p-IKKβ, and p-IκBα, and significantly (*p* < 0.05) enhanced expression of ZO-1 and occludin, as evaluated by Western-blot analysis of these proteins expressed in colonic tissue. The EPS produced by *L. plantarum* NMGL2 alleviated IBD by suppressing the NF-κB signaling pathway, suggesting its potential as a functional food agent in the prevention of IBD.

## Introduction

Inflammatory bowel disease (IBD) is a chronic intestinal disease without a particular etiology, and it is generally caused by various environmental triggers, genetic factors, immunoregulatory defects, and microbial exposure (Burisch et al., 2013; Hanauer, 2006). Symptoms of IBD usually present as abdominal pain, diarrhea, rectal bleeding, body weight loss, fever, and fatigue, which significantly affect human health (Emerson et al., 2021; Strober et al., 2007). With the worldwide increasing incidence of IBD, it is becoming urgently challenging to find alternative treatments for IBD to replace partially common drugs such as 5-aminosalicylate, sulfasalazine (SF) and corticosteroids, which have significant side effects and are expensive (Bernstein, 2017; Zhang et al., 2020; Yang et al., 2022).

Probiotics have been used to relieve the symptoms of IBD by reducing oxidative stress, altering cytokine secretion, repairing the colonic epithelial barrier, restoring the expression of tight junction (TJ) - associated proteins, and restoring gut microbiota (Roy and Dhaneshwar, 2023). Administration of the skimmed milk containing *Lacticaseibacillus paracasei* CCFM1222 was shown to regulate the TLR4/MyD88/NF-κB and Nrf2 signaling pathways in colitis mice and alter the gut microbiota, which indicated that *Lacticaseibacillus paracasei* CCFM1222 had certain anti-inflammatory effect (Guo et al., 2024). Administration of a glycated conjugate of whey protein and galactose with *Lactobacillus gasseri* 4M13 had protective effects on the intestine against colitis and maintains the immune balance (Jeong et al., 2022). As an important facultative heterofermentative species of lactic acid bacteria (LAB), *Lactiplantibacillus plantarum* (*L. plantarum*) is widely present in dairy products, fermented foods, human mouth, intestinal tract, and stools, etc. (Sabo et al., 2014). *L. plantarum* ZS62 has been shown to play a positive role in preventing inflammation by modulating oxidative stress and immune response (Pan et al., 2021).

Exopolysaccharides (EPSs) generated by LAB have raised increasing concerns in relieving IBD due to their beneficial effects on the gastrointestinal tract (Oerlemans et al., 2021) and diverse bioactivities such as anti-tumour (Wang et al., 2014), anti-oxidation (Zhang et al., 2013), and immunomodulatory activities (Saadat et al., 2019). These polymers can be homo- or heteropolysaccharides (Korcz and Varga, 2021) which are produced in the form of capsule tightly bound to the cell surface, or as slime secreted into the surrounding medium (Rajoka et al., 2020; Lee et al., 2016). A previous study reported that the EPS produced by *L. plantarum* NCU116 could protect the colon from inflammation through activation of the STAT3 signaling pathway (Zhou et al., 2018). Moreover, Li et al. (2021) reported that the EPS produced by *Lacticaseibacillus rhamnosus* GG could protect against intestinal oxidative damage and apoptosis.

Previously in our laboratory, an EPS-producing *L. plantarum* NMGL2 with good tolerance to low temperature and acid stress was isolated and identified from traditional fermented dairy cheese (Zhang et al., 2021) This EPS showed strong resistance to human gastrointestinal fluids and beneficially influenced the fecal microbiota diversity (Yao et al., 2022). The current study was conducted to investigate the relieving effects of the EPS from *L. plantarum* NMGL2 on dextran sulfate sodium (DSS)-induced IBD in mice and its underlying mechanism by detecting its anti-inflammatory, anti-oxidative, and intestinal barrier effects. This study would advance understanding of the mechanism of the EPS obtained from *L. plantarum* NMGL2 in the amelioration of IBD.

## Materials and methods

### Preparation of EPS samples

The EPS was produced using Yao et al. (2022) method. Briefly, *L. plantarum* NMGL2 (Dairy Laboratory of Beijing Technology and Business University of China) was activated at 37°C for 18 h in MRS broth medium by three consecutive transfers and was inoculated (3%, v/v) into liquid skim milk medium containing 10% (w/v) skim milk powder at 37°C for 24 h. The EPS was obtained by ethanol precipitation and purification by DEAE-Sepharose Fast Flow ion exchange column for the following tests.

### Animal experiment

Animal experiment was conducted using 48 six-week-old healthy male BALB/C mice (SPF Biotechnology Co., Ltd., Beijing, China) strictly in accordance with the provisions and general proposals of China’s laboratory animal management regulations. The mice were initially acclimatized in sawdust-lined plastic cages for 7 days at 22–24°C under a 12 h light/dark cycle with free access to food and water. The experiment was conducted randomly: normal control group (NC), model group (M), positive control group (P, 0.45 g/kg SF, Beijing Boosen Biotechnology Co., Ltd), low-dose EPS group (EPS-L, 20 mg/kg EPS), medium-dose EPS group (EPS-M, 40 mg/kg EPS), and high-dose EPS group (EPS-H, 80 mg/kg EPS). Except for NC group, IBD in mice was established by giving 5% DSS (MP Biomedicals, LLC, France) in drinking water (w/v) for 14 days. After IBD model was established, the NC and M groups were given the same amount of normal saline (10 mL/kg) for 7 days. The mice were administered or injected with 20 mg/kg EPS, 40 mg/kg EPS, and 80 mg/kg EPS or 0.45 g/kg SF (according to the body weight) for 7 days (once a day). At the end of 7 days treatment, the mice were anesthetized with 1% sodium pentobarbital, then dislocated and executed. The colons and contents were immediately removed and frozen under aseptic manipulation.

### Assessment of disease activity index

The disease activity index (DAI) score (from 0 to 4) was evaluated based on the method of Murano et al. (2000) with some changes. During the induction and treatment periods, the body weight loss (0: none, 1: 0–5%, 2: 5–10%, 3: 10–15%, 4: ≥15%), stool consistency (0: normal, 1–3: loose, 4: diarrhoea), and stool Haemoccult positivity (0: negative, 1–3: positive, 4: stool Haemoccult positivity) were recorded regularly every day. The DAI score was the sum of the body weight loss, stool consistency, and stool Haemoccult positivity scores divided by 3.

### Histopathological analysis

The extent of colon damage was quantified by histopathological analysis including the extent and depth of colon lesions. The coefficient of the colon was equal to the weight of the colon divided by the body weight of the mice. The histological scoring system including the extent (0: none, 1: mucosa, 2: submucosa, 3: muscularis, 4: transmural) and depth (0: normal, 1: 1–25%, 2: 26–50%, 3: 51–75%, 4: 76–100%) of colon lesions was modified based on a method previously described by Dieleman et al. (1998). The score for colonic histology was viewed as the sum of the score for the extent of colonic lesions and the score for the depth of colonic lesions. The colon histopathology was observed based on the method of Chung et al. (2021). Briefly, the colon tissue was removed by dissection in a sterile environment and its length was measured. Then the colon sections (about 1 cm at the distal end of the colon) were washed with saline, stabilized in 4% paraformaldehyde for 24 h, and embedded in paraffin. The sections were cut (5 μm) and stained with hematoxylin and eosin for 5 min. Histopathology changes were observed with photomicrography system (MF43, Mingmei Photoelectric Technology Co., Ltd., Guangzhou, China).

### Cytokine assay

Blood samples were obtained by the removal of the eyeball method and were left at 25°C for 2–4 h. The serum samples were obtained through refrigerated centrifugation for 15 min at 3,000 rpm and kept at −20°C for testing. The colon tissues were ground with pre-cooled lysate and centrifuged at 14,180 × g for 10 min at 4°C to obtain the supernatant for testing. The levels of cytokines interleukin-1β (IL-1β), tumor necrosis factor-α (TNF-α), and interleukin-10 (IL-10) in mice serum and colon tissue were measured by the enzyme-linked immunosorbent assay (ELISA) kit (Raybiotech, Guangzhou, China). All processes were strictly conducted according to the manufacturer’s specifications. Briefly, 100 μL of sample was added to each well and incubated for 2.5 h at room temperature. Then the liquid in the wells was discarded, shaken dry, and washed. One hundred microliters of antibody working solution was added to each well and incubated for 1 h at room temperature. After discarding the solution, 100 μL of enzyme conjugate working solution was added to each well and incubated for 45 min at room temperature. One hundred microliters of substrate solution was added to each well and incubated for 30 min at room temperature under dark conditions. Fifty microliters of stop solution was added to each well, and optical density of each well was immediately measured at 450 nm.

### Assay of oxidative stress

The colon tissue and normal saline were homogenized at a ratio of 1:9 (g:mL) under the condition of ice water bath and centrifuged at 2,500 rpm/min for 10 min. The supernatant was collected, and the levels of nitric oxide (NO), glutathione (GSH), malondialdehyde (MDA) and superoxide dismutase (SOD) in the colon tissue were determined according to the manufacturer’s specifications using the matching assay kits (Nanjing Jiancheng Technology Co., Ltd., Nanjing, China). Briefly, for NO measurement, 20 μL of reagent I and 100 μL of reagent II were added to 300 μL of homogenised supernatant and mixed well. After centrifugation, 160 μL of the supernatant was taken and mixed with the colour rendering agent. After 15 min, the optical density value of each well was measured at 550 nm. For MDA measurement, 100 μL of the test sample was mixed with 100 μL of reagent I, 3 mL of reagent II and 1 mL of reagent III and heated in a water bath for 40 min at 95°C. Then cooled under running water and centrifuged to determine the optical density at 532 nm. For GSH measurement, 2 mL of reagent I and 0.5 mL of homogenate supernatant were mixed and centrifuged at 3,500–4,000 rpm/min for 10 min. Then 1 mL of the supernatant was taken for colour development and kept for 5 min to be used for optical density determination at 420 nm. For SOD measurement, 50 μL of the sample, 1 mL of the reagent I, 100 μL of the reagent II, 100 μL of the reagent III, and 100 μL of the reagent IV were mixed well and heated 37°C for 40 min. Then 2 mL of colour rendering agent was added and the optical density value were determined at 550 nm.

### Western-blot analysis of the NF-κB pathway proteins

Western-blot analysis of the NF-κB pathway proteins in colonic tissue was performed by the modified method of Tian et al. (2021). Briefly, the protein was measured by applying a BCA protein detection kit (Beijing Solaibao Technology Co., Ltd., Beijing, China), and then the gross protein in the colon tissue was separated by 10% sodium dodecyl sulfate (SDS) polyacrylamide gel electrophoresis. The isolated protein was then transported to 0.22 μm polyvinylidene difluoride (PVDF) membrane, which was placed in the blocking solution [Tris-buffered saline with Tween (TBST) containing 5% skimmed milk powder] at 25°C for 30 min. The primary antibodies diluted with the blocking solution including NF-κB p65 (1:1000, Abcam), p-IKBα (1:1000, Beijing Boosen Biotechnology Co., Ltd.), p-IKKβ (1:1000, Beijing Boosen Biotechnology Co., Ltd.), and GAPDH (1:500, Abcam) were added and incubated at 25°C for 1 h. The primary antibody, secondary antibody and enhanced chemiluminescence solution were dropped onto the PVDF membrane. The signal was acquired using a chemiluminescence imager (ChemiScope 3300 Mini, Shanghai Qinxiang Scientific Instrument Co., Ltd., Shanghai, China), and the values of protein bands were analyzed (ImageJ software). Quantitative data were normalized to internal controls (GAPDH) and expressed as relative protein expression.

### Immunofluorescence assay

Paraffin-embedded colon tissue slices were prepared to 5 μm thickness. Slices were dewaxed with xylene and hydrated by gradient alcohol immersion (100, 95, 70%). Antigen repair was performed using citrate buffer, phosphate buffer solution (PBS) washed 3 times for 2 min at a time, and sealed with 5% bovine serum albumin (BSA) at 25°C for 1 h. One hundred microliters of diluted primary antibody was added dropwise at the concentration of NF-κB p65 (1:400), ZO-1 (1:400), and occludin (1:200), and incubated overnight in the shade at 4°C. Each section was washed 3 times with PBS solution and incubated with diluted Alexa Fluor 594-labeled goat anti-rabbit IgG at 25°C and shielded from light for 1 h. Afterwards, the slices were washed again by using PBS solution. DAPI was added to the sealer, mixed well, and the sealer was added to the slide, covered with a coverslip, and left at least 10 min at 25°C and shielded from light. Sealing, observation, and optical density value analysis of 400× images using image-Pro 6.0.

### Statistical analysis

The experimental results were shown as mean ± standard deviation at least three independent experiments. Normality data was evaluated. Differences among groups were assessed and analyzed by one-way ANOVA and Duncan’s multiple range test with SPSS 20.0 (SPSS Inc., Chicago, IL, United States). The *p* < 0.05 was considered statistically significant.

## Results

### Effect of the EPS on the body weight and DAI of IBD mice

**Figure 1:**
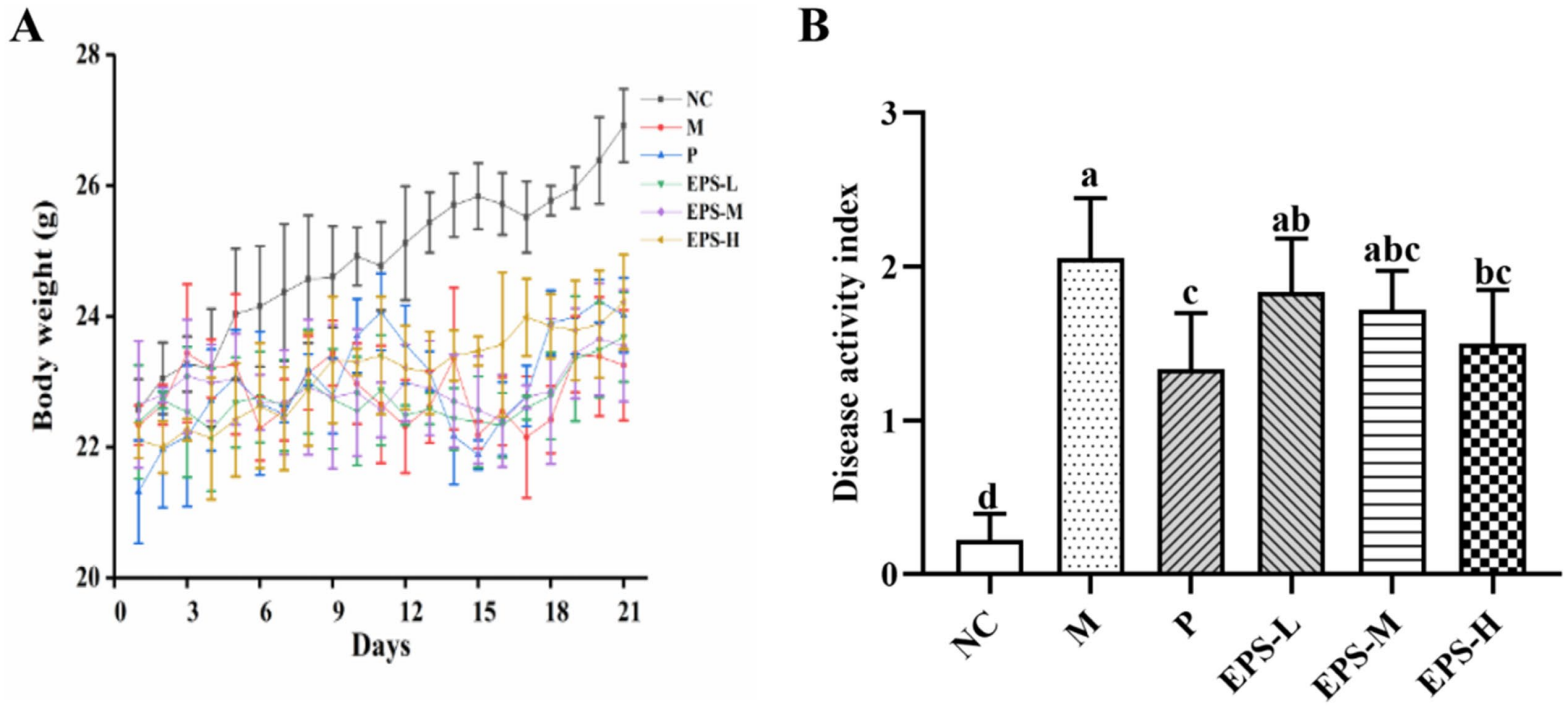
Effect of the EPS produced by *L. plantarum* NMGL2 on DSS-induced IBD symptoms in mice as investigated in the following groups: **(A)** body weight; **(B)** DAI score; NC: normal control; M: 5% DSS; P: 5% DSS + SF; EPS-L: 5% DSS + 20 mg/kg EPS; EPS-M: 5% DSS + 40 mg/kg EPS; EPS-H: 5% DSS + 80 mg/kg EPS. The results were presented as the mean ± standard deviation (SD) (*n* = 6 per group). Values with different superscript letters (a–d) significantly differed at *p* < 0.05.

Effect of the EPS generated by *L. plantarum* NMGL2 on the body weight and DAI of the IBD mice model established by 5% DSS was evaluated. As depicted in Figure 1A, the mice in group NC grew well with a steady increase in body weight during the 21-day experimental period (Figure 1A). After DSS induction, the body weight of mice in the NC, M, P, EPS-L, EPS-M, and EPS-H groups were 25.70 ± 0.49, 23.35 ± 1.08, 22.17 ± 0.74, 22.43 ± 0.47, 22.70 ± 0.71, and 23.40 ± 0.38 g, respectively. After treatment with normal saline (NC and M group), EPS (EPS-L, EPS-M, and EPS-H group), or SF (P group), the body weight of mice in the NC, M, P, EPS-L, EPS-M, and EPS-H groups were 26.92 ± 0.56, 23.25 ± 0.84, 24.02 ± 0.57, 23.68 ± 0.69, 23.55 ± 0.85, and 24.22 ± 0.73 g, respectively. The DAI scores of the groups NC, M, P, EPS-L, EPS-M, and EPS-H were assayed at the last day of the experiment (Figure 1B). The DAI scores were significantly (*p* < 0.05) lower in the NC group than in the M group (2.06 ± 0.39). After SF treatment, group P (1.33 ± 0.37) showed lower DAI scores compared with group M (*p* < 0.05). After high-dose EPS treatment, group EPS-H (1.50 ± 0.35) also showed lower DAI scores compared with group M (*p* < 0.05). No significant differences (*p* > 0.05) in DAI scores were found among the three EPS groups. No significant differences in DAI scores were also found between the groups EPS-H and P (*p* > 0.05).

### Effect of the EPS on the colon injury of IBD mice

**Figure 2:**
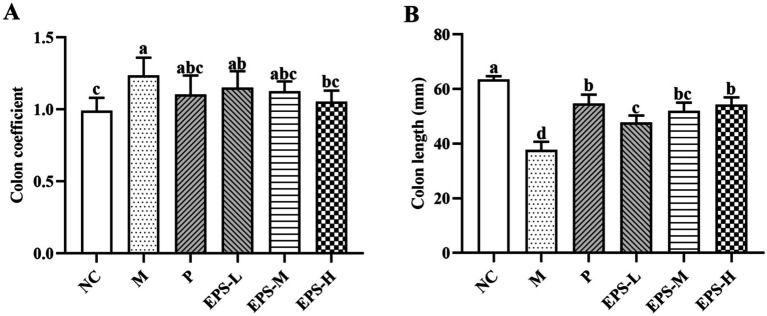
Effect of the EPS produced by *L. plantarum* NMGL2 on DSS-induced colon damage in IBD mice as investigated in the following groups: **(A)** colon coefficient; **(B)** colon length; NC: normal control; M: 5% DSS; P: 5% DSS + SF; EPS-L: 5% DSS + 20 mg/kg EPS; EPS-M: 5% DSS + 40 mg/kg EPS; EPS-H: 5% DSS + 80 mg/kg EPS. The results were presented as the mean ± standard deviation (SD) (*n* = 6 per group). Values with different superscript letters (a–d) significantly differed at *p* < 0.05.

Figure 2 shows the colon coefficient and colon length of the mice in the different experimental groups. M group (1.24 ± 0.14) had significantly higher colon coefficient (*p* < 0.05) than NC group (0.99 ± 0.09), while those of P (1.10 ± 0.13), EPS-L (1.15 ± 0.11), EPS-M (1.13 ± 0.07), and EPS-H (1.05 ± 0.07) groups fell in between, and had no significant differences among them (*p* > 0.05) (Figure 2A). Similarly, NC had the longest colon length (63.50 ± 1.22) and M the shortest (36.83 ± 3.76 mm), while those of P (54.67 ± 3.78 mm), EPS-L (47.67 ± 3.33 mm), EPS-M (52.00 ± 3.85) and EPS-H (56.33 ± 5.35 mm) fell in between (Figure 2B), and the EPS displayed dose-dependent effect on recovering the colon length of the DSS-induced IBD mice.

**Figure 3:**
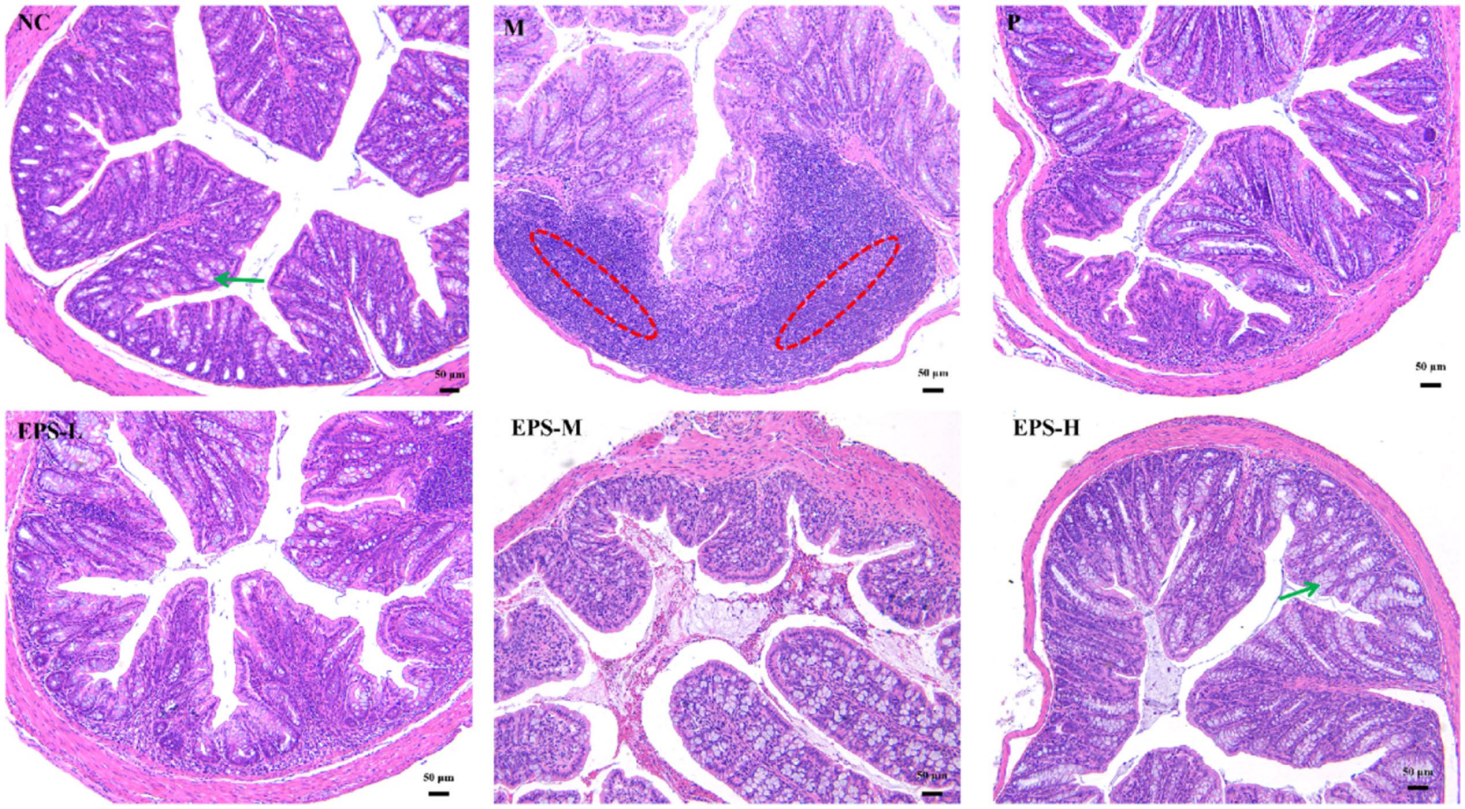
Histopathological observation of colon sections stained with hematoxylin and eosin in IBD mice as affected by the EPS produced by *L. plantarum* NMGL2 (*n* = 3 per group). NC: normal control; M: 5% DSS; P: 5% DSS + SF; EPS-L: 5% DSS + 20 mg/kg EPS; EPS-M: 5% DSS + 40 mg/kg EPS; EPS-H: 5% DSS + 80 mg/kg EPS. The red circle indicate inflammation and the green arrows indicate goblet cells. Scale bars represent 50 μm.

Figure 3 shows the histopathological assessment of the colon tissue of the mice from the different groups. For the NC mice, the colon structure was intact with neatly arranged cup cells and no significant immune cell infiltration. In contrast, the colon mucosal structure of the M group mice was severely damaged with hemorrhage and edema in the submucosa and epithelial cell detachment. After SF or different doses of EPS intervention, the colon mucosal structures of mice in the P, EPS-L, EPS-M and EPS-H groups were restored to some extent and the degree of inflammatory infiltration was alleviated. In addition, the EPS-H group (80 mg/kg) showed a better alleviating effect on inflammation than the EPS-L group (20 mg/kg).

**Figure 4:**
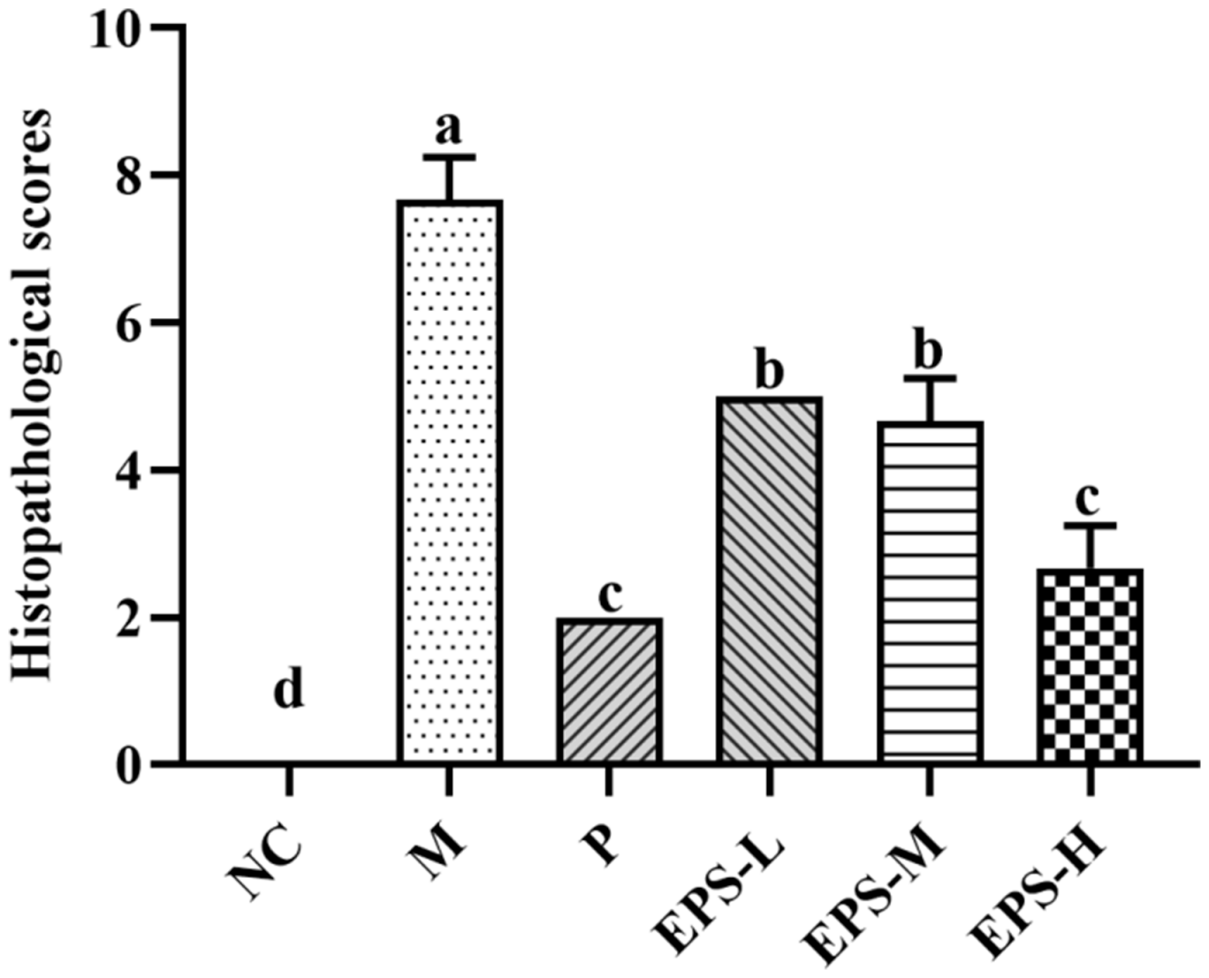
Effect of the EPS produced by *L. plantarum* NMGL2 on DSS-induced colon histological score in IBD mice as investigated in the following groups: NC: normal control; M: 5% DSS; P: 5% DSS + SF; EPS-L: 5% DSS + 20 mg/kg EPS; EPS-M: 5% DSS + 40 mg/kg EPS; EPS-H: 5% DSS + 80 mg/kg EPS. The results were presented as the mean ± standard deviation (SD) (*n* = 3 per group). Values with different superscript letters (a–d) significantly differed at *p* < 0.05.

The histopathological observation of the colon was consistent with the result of the histological scores of the colon (Figure 4). The histopathological scores of the groups containing EPS produced by *L. plantarum* NMGL2 were all significantly (*p* < 0.05) lower than group M. No significant (*p* > 0.05) differences in histopathological scores were observed between group P and group EPS-H.

### Effect of the EPS on the levels of inflammatory cytokines and oxidative stress of IBD mice

**Figure 5:**
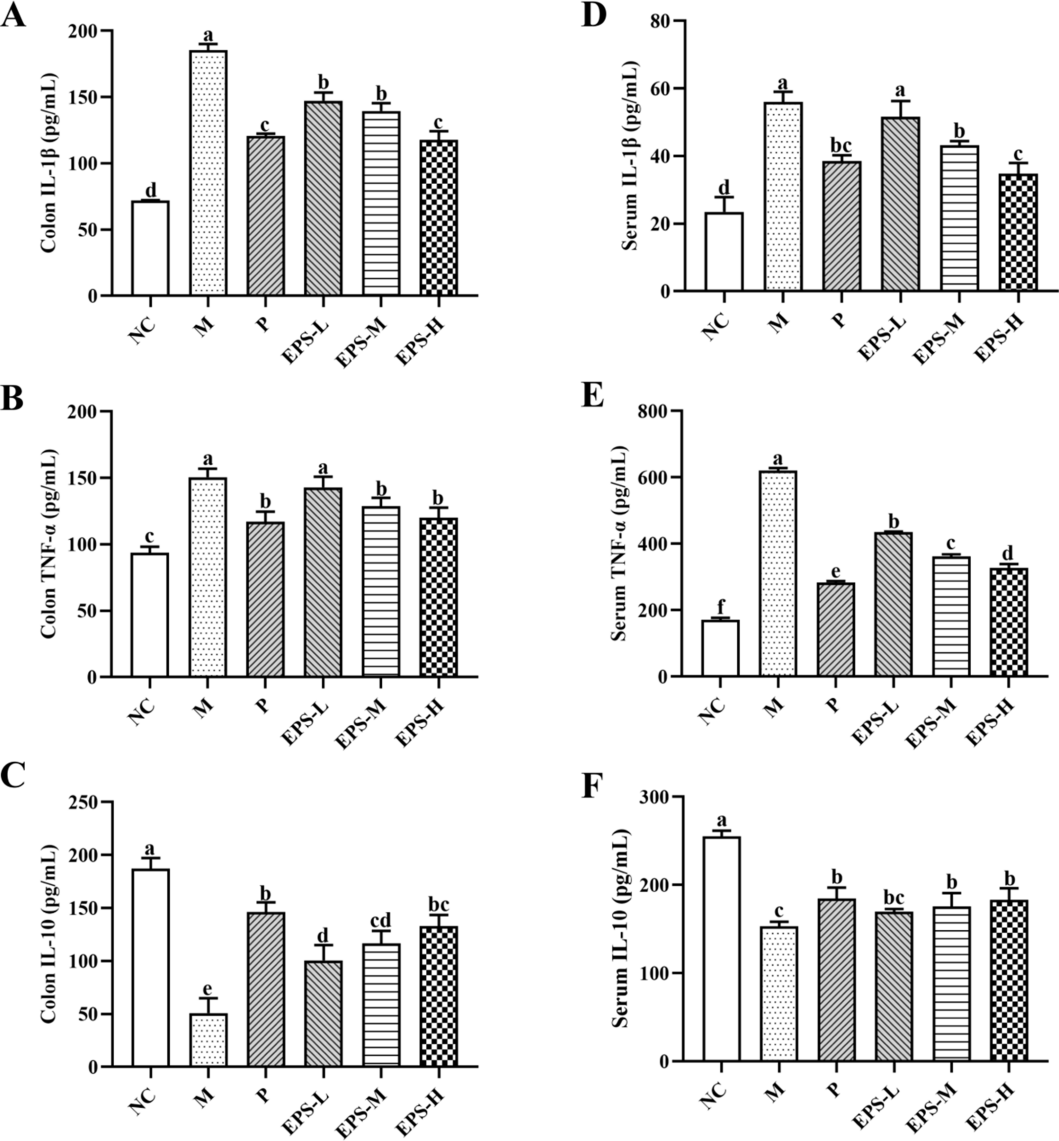
Effect of the EPS produced by *L. plantarum* NMGL2 on DSS-induced cytokine levels of colon tissue and serum in IBD mice as investigated in the following groups: **(A)** colon IL-1β; **(B)** colon TNF-α; **(C)** colon IL-10; **(D)** serum IL-1β; **(E)** serum TNF-α; **(F)** serum IL-10; NC: normal control; M: 5% DSS; P: 5% DSS + SF; EPS-L: 5% DSS + 20 mg/kg EPS; EPS-M: 5% DSS + 40 mg/kg EPS; EPS-H: 5% DSS + 80 mg/kg EPS. The results were presented as the mean ± standard deviation (SD) (*n* = 3 per group). Values with different superscript letters (a–e) significantly differed at *p* < 0.05.

Effect of the EPS on the inflammatory cytokine contents in the serum and colon tissues of mice is presented in Figure 5. Comparing with group NC, group M had significantly increased (*p* < 0.05) contents of pro-inflammatory cytokines IL-1β and TNF-α in serum and colon, but significantly reduced (*p* < 0.05) content of anti-inflammatory cytokine IL-10. Treatment with SF or different doses of EPS significantly decreased (*p* < 0.05) the contents of TNF-α and IL-1β, and significantly increased (*p* < 0.05) the level of IL-10, compared with group M. Furthermore, group EPS-H (80 mg/kg) showed a better treatment effect than group EPS-L (20 mg/kg).

**Figure 6:**
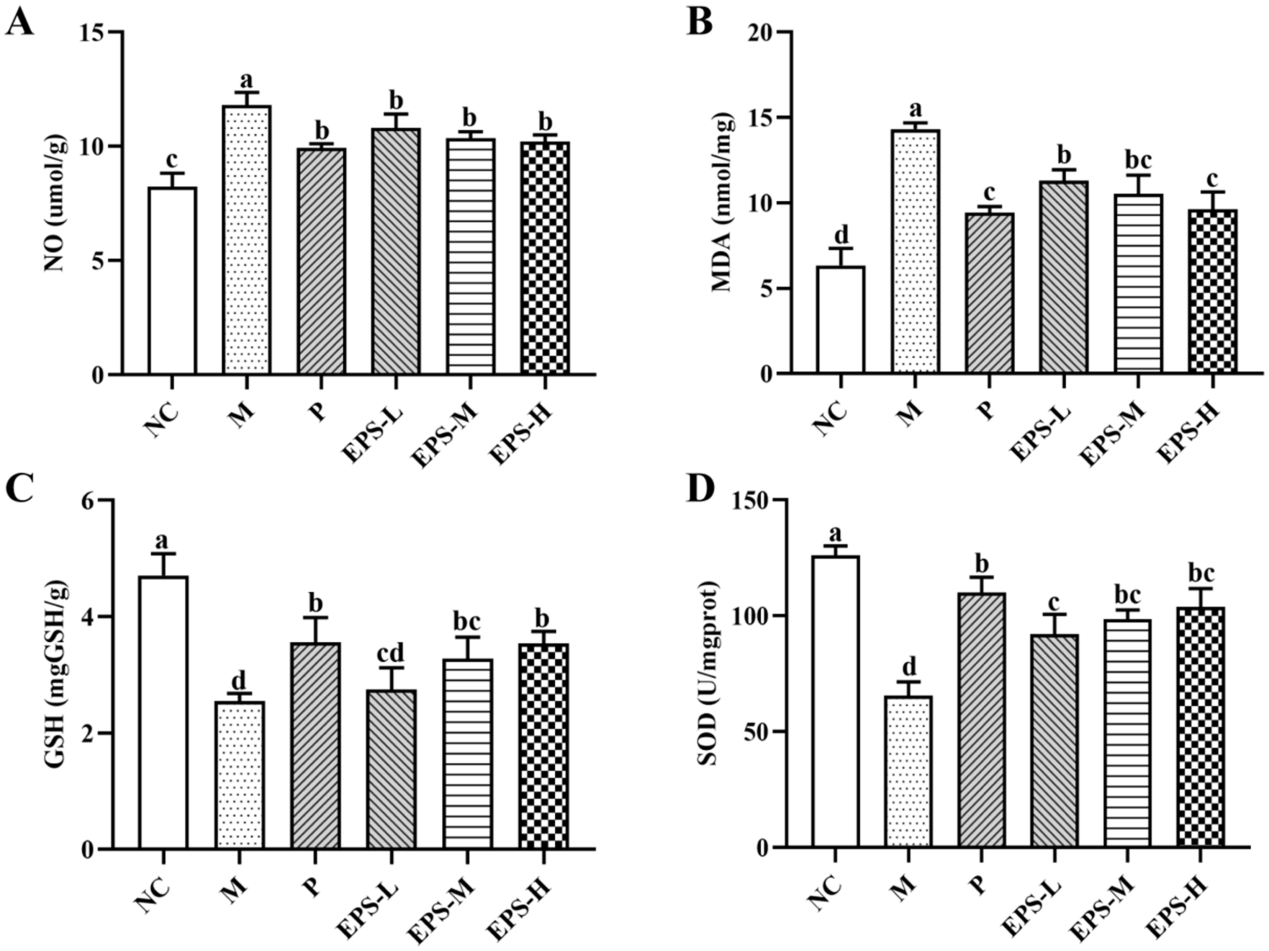
Effect of the EPS produced by *L. plantarum* NMGL2 on DSS-induced antioxidant activity in IBD mice as investigated in the following groups: **(A)** NO; **(B)** MDA; **(C)** GSH; **(D)** SOD; NC: normal control; M: 5% DSS; P: 5% DSS + SF; EPS-L: 5% DSS + 20 mg/kg EPS; EPS-M: 5% DSS + 40 mg/kg EPS; EPS-H: 5% DSS + 80 mg/kg EPS. The results were presented as the mean ± standard deviation (SD) (*n* = 3 per group). Values with different superscript letters (a–d) significantly differed at *p* < 0.05.

Figure 6 shows the antioxidant effect of the EPS on DSS-induced IBD in mice with different treatments. The contents of NO, MDA, GSH, and SOD in colon homogenate of the group M mice were determined to be 11.82 ± 0.54 umol/g, 14.33 ± 0.37 nmol/mg, 2.54 ± 0.14 mg/g, and 65.61 ± 5.96 U/mg, respectively. After different doses of EPS intervention for 21 days, the NO and MDA contents were decreased, but the GSH content and SOD activity were increased. Group EPS-H exhibited better anti-oxidative effect than EPS-M and EPS-L.

### Regulatory effect of the EPS on the NF-κB signaling pathway to alleviate intestinal barrier damage in IBD mice

**Figure 7:**
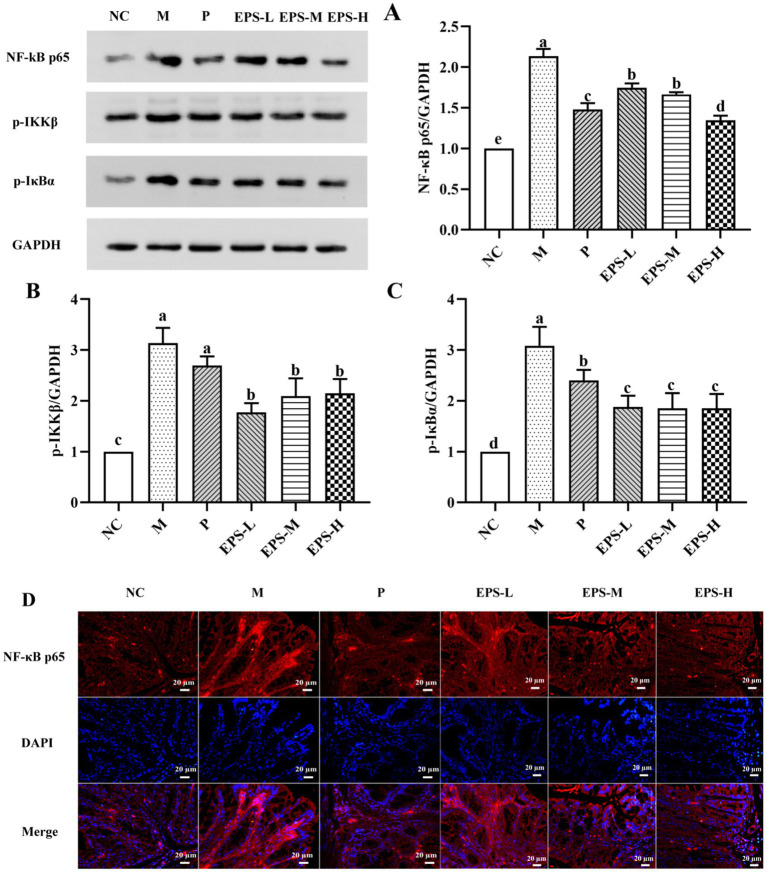
Inhibitory effect of the EPS produced by *L. plantarum* NMGL2 on DSS-induced NF-κB signaling pathway in IBD mice as investigated in the following groups: **(A)** NF-κB p65; **(B)** p-IKKβ; **(C)** p-IκBα; **(D)** Immunofluorescence analysis of NF-κB p65 in colon mucosa; NC: normal control; M: 5% DSS; P: 5% DSS + SF; EPS-L: 5% DSS + 20 mg/kg EPS; EPS-M: 5% DSS + 40 mg/kg EPS; EPS-H: 5% DSS + 80 mg/kg EPS. The results were presented as the mean ± standard deviation (SD) (*n* = 3 per group). Values with different superscript letters (a–e) significantly differed at *p* < 0.05. Scale bars represent 20 μm.

To further study the mechanism of effect of the EPS on IBD mice, changes of protein expression relevant to the NF-κB signaling pathway were evaluated. As shown in Figure 7, the NF-κB inflammatory signaling pathway was enabled in the M group of mice as evidenced by their dramatically increased expression of several key proteins such as NF-κB p65, p-IKKβ and p-IκBα, comparing with those in the NC group. Treatment with different doses of EPS significantly down-regulated (*p* < 0.05) expression of these proteins. These were consistent with the results of immunofluorescence, which showed changes of fluorescence intensity corresponding to the protein expression. Specially, NF-κB p65 in the M group showed the highest fluorescence intensity, and it became weaker after treatment with different doses of EPS, confirming the effectiveness of the EPS intervention to alleviate the severity of IBD.

**Figure 8:**
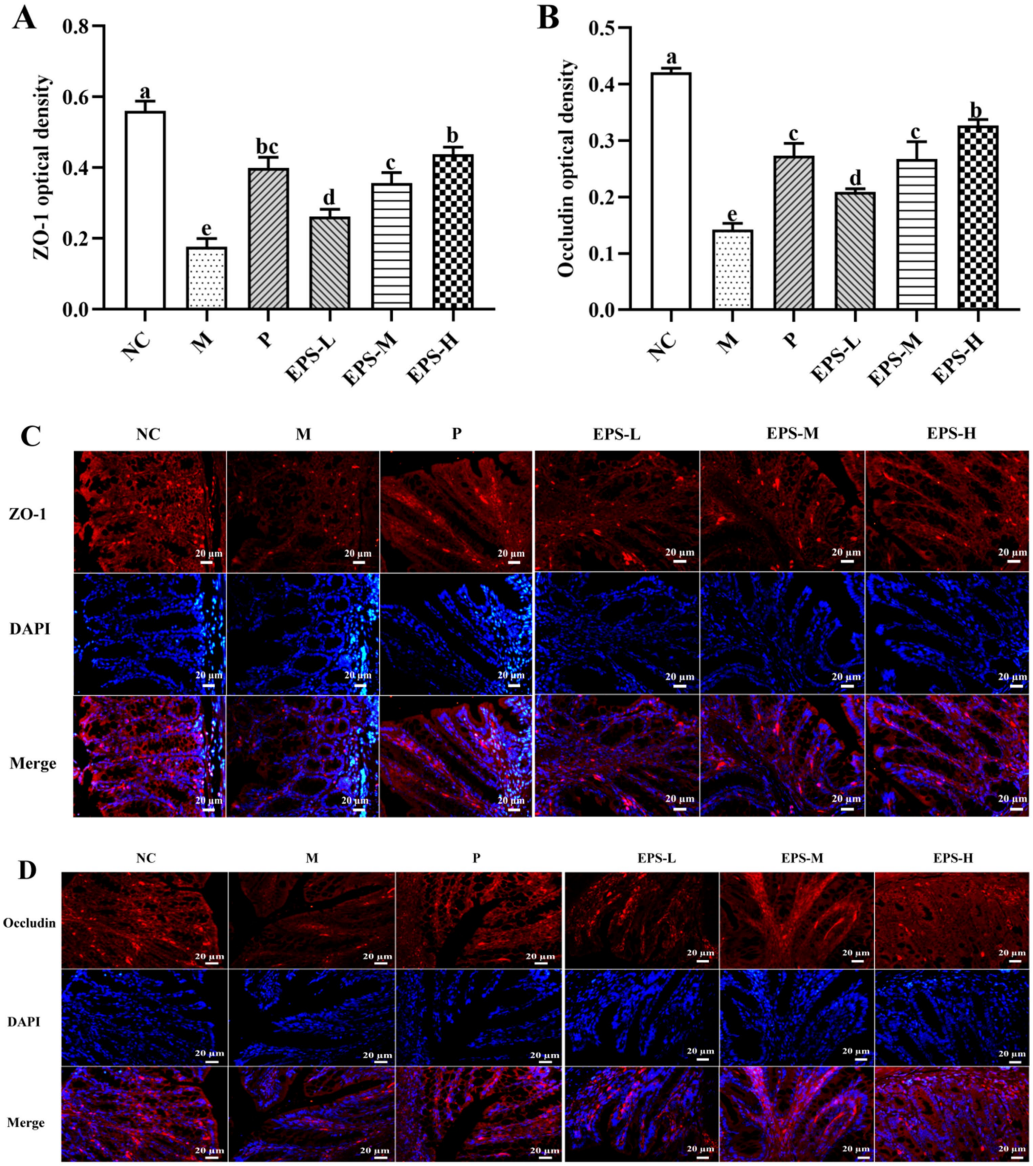
Effect of the EPS produced by *L. plantarum* NMGL2 on DSS-induced tight junction proteins content in IBD mice as investigated in the following groups: **(A)** optical density analysis of ZO-1 protein; **(B**) optical density analysis of occludin protein; **(C)** immunohistochemical staining of ZO-1 protein; **(D)** immunohistochemical staining of occludin protein; NC: normal control; M: 5% DSS; P: 5% DSS + SF; EPS-L: 5% DSS + 20 mg/kg EPS; EPS-M: 5% DSS + 40 mg/kg EPS; EPS-H: 5% DSS + 80 mg/kg EPS. The results were presented as the mean ± standard deviation (SD) (*n* = 3 per group). Values with different superscript letters (a–e) significantly differed at *p* < 0.05. Scale bars represent 20 μm.

Further investigation was also performed on the expression of TJ proteins, important factors contributing to intestinal integrity, as affected by treatment of the IBD mice with the EPS. As depicted in Figure 8, there was significant expression of ZO-1 and occludin proteins in the NC group, but significantly (*p* < 0.05) decreased expression of these proteins in the M group. Intervention with the EPS resulted in enhanced levels of TJ proteins in colon luminal epithelial cells and crypt epithelial cells of IBD mice, and the EPS-H (80 mg/kg) group of IBD mice showed higher enhancement of TJ proteins expression. Immunofluorescence imaging of these samples confirmed the effectiveness of the EPS to increase TJ proteins expression between the epithelial cells, as shown by the corresponding increased density of fluorescent rings when compared with those of the M group. There were significantly decreased (*p* < 0.05) contents of ZO-1 and occludin in the samples from the M group, corresponding to the reduced fluorescence intensity, which could be attributed to the colon damage such as necrosis and shedding of epithelial cells in the IBD mice.

## Discussion

IBD is a chronic intestinal disease characterized by abdominal pain, diarrhea, rectal bleeding, body weight loss, fever, and fatigue (Emerson et al., 2021; Strober et al., 2007). It is crucial to seek effective natural alternative treatments for IBD to replace partially common drugs such as 5-aminosalicylate, sulfasalazine (SF) and corticosteroids, which have significant side effects and are expensive (Bernstein, 2017; Zhang J. et al., 2020; Yang et al., 2022). Biological drugs such as antibodies adalimumab, golimumab, and vedolizumab are approved by the National Institute for Health and Care Excellence (NICE) for the treatment of inflammation of colitis (Tamilarasan et al., 2019). Furthermore, Drugs, mainly aminosalicylates, corticosteroids, and live probiotics can be delivered to the inflammatory sites in the gut through smart bionanomaterials such as smart hydrogels, nanoparticles, and nanofibers, which respond to external stimuli and can be used for the treatment and diagnosis of IBD (Stojanov and Berlec, 2024). In the current study, we found that the symptoms of DSS-induced IBD mice were alleviated after *L. plantarum* NMGL2 EPS treatment. The results suggested that the EPS could ameliorate IBD by inhibiting inflammation, relieving oxidative stress, and strengthening intestinal mucosal barrier. The effect of EPS on the symptoms of DSS-induced IBD mice could be investigated by assaying the relevant indicators of clinical manifestations and pathological features. The results of this study indicated that EPS intervention could suppress body weight loss, improve DAI scores, inhibit the shortening of colon length, and strengthen the colonic mucosal structure. Previously, the DSS-induced IBD mice showed shortened colon, acute mucosal injury, inflammatory cell immersion, crypt phagocytosis, and higher histological scores (Zhang Y. et al., 2020). The EPS from *Lacticaseibacillus rhamnosus* ZFM231 was reported to recover the body weight loss, colon length, DAI score, and the degree of colon damage in DSS-induced colitis model (Wan et al., 2022). In addition, previous studies reported that the body weight loss was also found in mice with DSS-induced colitis models (Su et al., 2022) The body weight loss and colon length shortening of DSS-induced mice could be prevented to some extent by treatment with the exopolysaccharide produced by *L. plantarum*-12 (LPEPS) (Ma et al., 2021). The similar effect of the EPS obtained from *L. plantarum* NMGL2 indicated potential of this EPS to be a promising food component for treatment of IBD.

Unbalanced production of pro-inflammatory and anti-inflammatory cytokines contributed to inflammation and immune dysfunction in IBD patients and animals with colitis (Soufli et al., 2016). TNF-α promoted T cell proliferation and differentiation to disrupt the intestinal barrier and accelerate intestinal inflammation, and IL-1β played a vital role in mediating the ongoing inflammatory response in the intestine (Zhang et al., 2019). Increased secretion of TNF-α resulted in an impaired intestinal barrier, and IL-1β was an essential cytokine that triggered local inflammation in the colonic mucosa (Shi et al., 2021) TNF-α and IL-1β were increased in IBD patients, while IL-10 mainly maintained intestinal homeostasis and down-regulated immune responses and pro-inflammatory mediators associated with innate and adaptive immune responses (Biasi et al., 2011). In the current study, we measured TNF-α, IL-1β, and IL-10 in the serum and colon tissue and found that the EPS significantly (*p* < 0.05) reduced the contents of TNF-α and IL-1β and increased the contents of IL-10. Moreover, higher doses of EPS (80 mg/kg) were more anti-inflammatory than lower doses of EPS (20 mg/kg and 40 mg/kg). Similarly, different doses of EPS obtained from *L. plantarum* YW11 were shown to decrease the TNF-α and IL-1β contents, and increase the IL-10 content in IBD model (Zhang M. et al., 2020). Ma et al. (2021) also found that LPEPS intervention significantly (*p* < 0.05) reduced the contents of pro-inflammatory cytokines including TNF-α and IL-1β, and increased the contents of anti-inflammatory cytokine IL-10 in DSS-induced colitis mice.

Oxidative stress, which was caused by an unbalance between excess reactive oxygen groups and the antioxidant defense systems (Gao et al., 2021) was thought to play a vital role in the induction and transmission of IBD (Li et al., 2017). Oxygen radicals contributed to increased emission of inflammatory agents, which not only induced direct epithelial injury, but also exacerbated oxidative stress, thus resulting in damage of the physical barrier by affecting the local epithelium (Shi et al., 2019). In the current study, NO and MDA levels were significantly enhanced (*p* < 0.05), but SOD activity and GSH levels were significantly decreased (*p* < 0.05) in IBD mice after DSS induction. The EPS intervention suppressed the elevated levels of NO and MDA, and increased the contents of GSH and SOD activity, indicating the effectiveness of the EPS in the prevention of IBD by relieving the oxidative stress. The EPS produced by two probiotic strains (*L. delbruckii* subsp. *bulgaricus* B3 and *L. delbruckii* subsp. *bulgaricus* A13) was also effective in ameliorating oxidative stress in colitis model (Sengul et al., 2011).

NF-κB pathway can be enabled by TNF-α, and NF-κB could facilitate production of TNF-α and IL-1β (Chen et al., 2019). In this study, treatment with the EPS significantly down-regulated (*p* < 0.05) the levels of NF-κB p65, p-IKKβ, and p-IκBα, indicating that the EPS alleviated IBD by the mechanism of regulation on NF-κB signaling pathway. Furthermore, increased expression of occludin and ZO-1 was found to enhance the intestinal barrier in DSS-induced colitis mice (Wang et al., 2018; Srutkova et al., 2015). Alteration of the colon structure, including lower expression of ZO-1 and occludin, was among the important factors contributing to intestinal barrier disruption in IBD patients (Zhang et al., 2019) Treatment with the EPS derived from *L. plantarum* NMGL2 of this study increased the levels of ZO-1 and occludin in IBD mice, which strengthened the intestinal mucosa and alleviated enteritis in mice. Previously, increased expression of occludin and ZO-1 was also observed in IBD model treated with the EPS from *L. plantarum* NCU116 (Zhou et al., 2018). Administration of the ropy EPS-producing strain of *Bifidobacterium longum* subsp. *longum* YS108R maintained the integrity of the mucosal barrier with enhanced expression of ZO-1 and occluding (Yan et al., 2019).

Overall, NF-κB played a crucial role in regulating the production of the pro-inflammatory cytokines involved in DSS-induced colitis mice and oxidative stress could impair the integrity of intestinal mucosa barrier (Aziz et al., 2024; Wu et al., 2020). The contents of pro-inflammatory cytokines were decreased and TJ proteins were enhanced after *L. plantarum* NMGL2 EPS treatment, which revealed the potential mechanism of *L. plantarum* NMGL2 EPS on ameliorating of the symptoms in DSS-induced colitis mice through the regulatory of inflammatory cytokines, suppression of oxidative stress, and increased TJ proteins. Group EPS-H and P had similar results in attenuating DSS-induced colitis in mice, which indicated the *L. plantarum* NMGL2 EPS might be a candidate for the treatment of inflammatory diseases.

## Conclusion

Intervention with the EPS from *L. plantarum* NMGL2 had remarkable therapeutic effects on DSS-induced IBD mice. Administration of the EPS could inhibit body weight loss, lower DAI scores, and inhibit colonic shortening. The EPS intervention also led to decreased pro-inflammatory (TNF-α and IL-1β) and increased anti-inflammatory (IL-10) contents, as well as favorable changes of antioxidant enzymes in the blood and colon tissue. Further down-regulated levels of NF-κB p65, p-IKKβ, and p-IκBα, and enhanced expression of ZO-1 and occludin in colon tissue indicated that the EPS alleviated IBD by suppressing the NF-κB signaling pathway. Hence, these findings suggest that the EPS from *L. plantarum* NMGL2 can serve as a promising supplement for the management of IBD.

## Data Availability

The raw data supporting the conclusions of this article will be made available by the authors, without undue reservation.
